# Improving stability and understandability of genotype-phenotype mapping in *Saccharomyces* using regularized variable selection in L-PLS regression

**DOI:** 10.1186/1471-2105-13-327

**Published:** 2012-12-08

**Authors:** Tahir Mehmood, Jonas Warringer, Lars Snipen, Solve Sæbø

**Affiliations:** 1 Biostatistics, Department of Chemistry, Biotechnology and Food Sciences, Norwegian University of Life Sciences, Ås, Norway; 2 Department of Animal and Aquaculture, Centre of Integrative Genetics (CIGENE), Ås, Norway; 3 Department of Cell- and Molecular Biology, University of Gothenburg, Gothenburg, Sweden

## Abstract

**Background:**

Multivariate approaches have been successfully applied to genome wide association studies. Recently, a Partial Least Squares (PLS) based approach was introduced for mapping yeast genotype-phenotype relations, where background information such as gene function classification, gene dispensability, recent or ancient gene copy number variations and the presence of premature stop codons or frameshift mutations in reading frames, were used *post hoc* to explain selected genes. One of the latest advancement in PLS named L-Partial Least Squares (L-PLS), where ‘L’ presents the used data structure, enables the use of background information at the modeling level. Here, a modification of L-PLS with variable importance on projection (VIP) was implemented using a stepwise regularized procedure for gene and background information selection. Results were compared to PLS-based procedures, where no background information was used.

**Results:**

Applying the proposed methodology to yeast *Saccharomyces cerevisiae* data, we found the relationship between genotype-phenotype to have improved understandability. Phenotypic variations were explained by the variations of relatively stable genes and stable background variations. The suggested procedure provides an automatic way for genotype-phenotype mapping. The selected phenotype influencing genes were evolving 29% faster than non-influential genes, and the current results are supported by a recently conducted study. Further power analysis on simulated data verified that the proposed methodology selects relevant variables.

**Conclusions:**

A modification of L-PLS with VIP in a stepwise regularized elimination procedure can improve the understandability and stability of selected genes and background information. The approach is recommended for genome wide association studies where background information is available.

## Background

The explosive growth of data describing the natural genetic and phenotypic variation within species, and the corresponding emergence of populations genomic [1] and population phenomics [2] as nascent fields of research demands new or improved methods for exploring genotype-phenotype relationships. In a recent proof of concept study, we introduced multivariate analysis in the form of Soft-Thresholding Partial Least Squares (ST-PLS) [3] for the mapping of genotype and phenotype interactions in the yeast, *Saccharomyces cerevisiae*[4], which has been at the center point for this development. Multivariate approaches have the potential to provide superior statistical power, increased interpretability of results and a deeper functional understanding of the genotype-phenotype landscape as it pays attention to relationships between multiple genotypes and multiple phenotypes, without producing an excessive number of hypotheses to test. Hence, it could provide decisive advantages over classical univariate analysis [5-9]. A caveat is the sensitivity of multivariate approaches to parameter estimation and this remains a serious challenge, partially because variables tend to show extensive collinearity, which can destroy the asymptotic consistency of the PLS estimators for univariate responses [10] and partially because signal-to-noise ratios are often low. A possible solution to the challenge of estimating parameters correctly is to guide these estimations by including background information on variable relationships at the modeling stage [11]. For genotype-phenotype mapping, such information could encompass the location of genes in the genome, the degree of shared regulatory elements, functional relatedness in terms of biochemical activity, molecular process or subcellular localization of gene products or physical interactions between gene-products. Also, for biological interpretation of the results, focus in relational studies is getting shifted from the selection of genes towards the selection of groups of genes listed as background information [12,13], but the selection of groups of genes can be missed if only few of the corresponding genes are significant [14]; hence a powerful structure extraction tool is required.

Indeed, the analytical implications of including background information to optimize parameter estimation in multivariate analysis, in the context of a three block Partial Least Squares based method named L-PLS regression, has recently been considered [15,16]. The mapping of genotype-phenotype relations through L-PLS requires a variable selection step. We recently [17] introduced a backward stepwise elimination procedure in PLS for identifying codon variations discriminating different bacterial taxa, where significant number of variables were eliminated at the cost of a marginal decrease in model accuracy. This approach was found to be superior to other multivariate procedures with respect to the understandability of the model and the consistency of the estimates. Here, we investigate whether inclusion of background information in the parameter estimation step of a multivariate analysis can increase the stability and understandability when applied in the context of mapping genotype-phenotype relationships in large data sets. Applying L-PLS and a stepwise backward elimination procedure, we find the inclusion of background information to enhance both stability and understandability in an automatic way and thus to constitute a promising way forward.

## Methods

### Approach

#### Data

##### Simulated data

To demonstrate the efficacy of the proposed procedure for variable selection when background information on the variables is available, simulation data from the following known model, also used by [15], is considered. *E*(***y***)=***XβX***_(*N*×*K*)_, having *Kx* vector follow the multivariate normal distribution with mean-vector *μ*=0 and covariance-matrix *Σ*_*x*_. Response vector ***y***_(*N*×1)_was assumed to follow a standard normal distribution, and joint distribution of $h={\left[x^{⊤}\;\;\tilde{y}\right]}^{⊤}$ is

(1) $$\;h=\left[\begin{array}{l} x\\ \tilde{y} \end{array}\right]∼\mathrm{MVN}\left(\left[\begin{array}{l} 0\\ 0 \end{array}\right],\left[\begin{array}{ll} \Sigma_{x} & \sigma_{x\tilde{y}}\\ \sigma_{x\tilde{y}}^{⊤} & 1 \end{array}\right]\right)=\mathrm{MVN}(0,\Sigma )$$

where $\sigma_{x\tilde{y}}$ is the covariances vector between ***x*** and $\tilde{y}$. By imposing a block diagonal structure on ***Σ***_*x*_groups of correlated x-variables were constructed by *L* blocks:

(2) $$\Sigma_{x}=\left[\begin{array}{lllll} \Sigma_{k_{1}} & 0 & \cdot & \cdot & 0\\ 0 & \Sigma_{k_{2}} & \cdot & \cdot & 0\\ \cdot & \cdot & \cdot \\ \cdot & \cdot & \cdot \\ 0 & 0 & \cdot & \cdot & \Sigma_{k_{L}} \end{array}\right]$$

Suppose *k*_*l*_ is the number of variables in group *l* (*l*=1,…,*L*). A total of *L*=14 groups of variables were simulated with group sizes *k*_1_,…,*k*_14_=[ 10,10,10,100,10,10,10,10,10,10,10,100,100,100 ], and uniform correlation structures in each $\Sigma_{k_{l}}$ were assumed internally with correlations equal to *ρ*_1_,…,*ρ*_14_=[0*.*5,0*.*5,0*.*5,0*.*2,0*.*…,0], respectively. As the defined correlation matrix (2) indicates variables in different groups that are uncorrelated, we also set the variance of each variable equal to 1, which makes the ***Σ***_*x*_ a correlation matrix. We assume all variables in each group were equally relevant for prediction, and the covariances used in the simulation were $\left[\sigma_{x\tilde{y}1},\cdots \;,\sigma_{x\tilde{y}14}]=[0.35,-0.3,0.25,0.2,0,\ldots ,0\right]$, and simulated response vector was transformed to a binary variable, coded as −1 and 1. Only the four first groups of variables were relevant for classification due to their non-zero values of the covariance with *y*. Since variables are grouped, variable grouping information was used as background information, and ***Z*** was coded as given in [15].

##### Real data

The data set is identical to that used in a recently conducted study [4], which did not utilize background information for parameter estimation. The data set contains 36 *Saccharomyces cerevisiae* strains, including the reference strain S288C [1,18]. Each strain has 16 chromosomes and a mitochondrial genome. In total 5791 protein-coding sequences, excluding dubious genes, were used as reference sequences. Each genome protein coding gene element, was converted into a vector of numeric features by sequence alignment of that element in a particular strain to the corresponding sequence element in the reference genome S288C. To achieve this, all reference sequences were first aligned against themselves, and for each reference sequence, the maximum alignment score, representing some coding gene of the S288C genome, was obtained. Then each individual *S. cerevisiae* genome was BLASTed against this reference set, using tblastx (http://blast.ncbi.nlm.nih.gov/Blast.cgi?CMD=Web&PAGE_TYPE=BlastHome). Hence for each genome sequence a maximum bit-score was obtained, providing a measure of to what extent the reference sequence was found in each individual genome. Since this score depends heavily on the length of the aligned sequences, numeric features were finally translated by Jukes-Cantor evolutionary distances. Numeric features were then assembled into a matrix ***X***_(*N*×*K*)_ with *N*=36 rows and *K*=5791 columns, one column for each reference sequence element. Data for each phenotype was assembled into a column vector ***y*** of length *N*=36, where the phenotype data were obtained by micro-cultivation of yeast strains in 10 different environments [2,19]. High density growth curves were parameterized [20] into measures of the reproductive rate (doubling time, Rate) and the reproductive efficiency (gain in population density given the available resources, Efficiency) and this results in 20 phenotype responses.

The genome of the yeast reference strain S288C is exceptionally richly annotated on a functional level, reflected in that data on functional relatedness in terms of shared Gene Ontology (GO) annotations [21] exists for a vast majority of its gene products. In its most extreme form, this annotation denotes genes as essential or not essential for viability. Furthermore, information on strain specific genetic variations with a potentially large effect on phenotypes has recently been extracted from population genomic data [1,2]. This information takes the form of the presence or absence of specific gene amplifications, reflecting potentially phenotype changing gain-of-function mutations, and the presence or absence of premature stop codons and frameshifts, reflecting likely loss-of-function mutations with potential negative effect on phenotypes. These information elements, taken together, serve as background information classifying genotypes in the study. Background information was assembled into a matrix ***Z***_(*L*×*K*)_ with *K*=5791 columns and *L*=51 rows, where each column represents a gene and each row represents a GO term (45) or a specific sequence variation (6). Each entry in ***Z***is denoted ‘1’ if the corresponding gene is associated with the respective GO term and denoted ‘0’ if not.

##### Genotype-phenotype relations

The data set consists of the column vector ***y***_(*N*×1)_representing each phenotype one at a time, the matrix ***X***_(*N*×*K*)_of genotypes based on Jukes-Cantor evolutionary distances and the matrix ***Z***_(*L*×*K*)_ of background information on genes containing annotated levels. To mine for relations between phenotypes and genotypes, we implemented an L-PLS approach [15] utilizing the background information in the modeling stage. We employed a PLS based algorithm for parsimonious variable selection [17] which is implemented here for multivariate feature selection in two stages, first for selection of genotype variables ***X***and then for the selection of background information variables ***Z***. In essence, we are looking for combinations of columns of ***X*** and rows of ***Z*** capable of explaining the variations in each ***y***, (see the Algorithm section for details).

### Algorithm

#### L-PLS supervised learning

The association between each phenotype vector ***y***and several genotype vectors ***X***, where background information ***Z***on ***X***is also given, was assumed to be explained by the linear model E(***y***)=***Xβ***where ***β***are the *K*×1 vector of regression coefficients. Least square fitting was no option because the number of samples (*N*=36) was much smaller than the number of features (*K*=5791). PLS resolves this by searching for a set of components, ‘latent vectors’, that performs a simultaneous decomposition of ***X*** and ***y***with the constraint that these components explain as much as possible of the covariance between ***X*** and ***y***. A large number of components indicates a complex relation is modeled and vice versa. However, in the standard implementation, it does not utilize background information ***Z*** in modeling. L-Partial Least Squares (L-PLS) [15,22] regression provides a way to include background information in the modeling, which is also similar to the bifocal-PLS of Eriksson *et al*[23]. The algorithm utilizes the NIPALS algorithm for the extraction of latent vectors, where relevancy of background information ***Z*** in modeling the (***yX***) relation is presented by *α*, as explained below. A large value of *α*indicates that ***Z***is relevant for explaining genotype-phenotype relations. The algorithm starts by centering as

(3) $$\begin{array}{lll} y_{0} & =y-1_{n}{\bar{y}}^{⊤}\; & \;\\ X_{00} & =X-1_{n}{\bar{x}}_{K}^{⊤}-{\bar{x}}_{N}1_{K}^{⊤}+\bar{\bar{x}}1_{N}1_{K}^{⊤}\; & \;\\ Z_{0} & =Z-\bar{z}1_{K}^{⊤}\;,\; & \; \end{array}$$

in the following manner where $\bar{y}$ is the column mean vector of ***y***, ${\bar{x}}_{K}$, ${\bar{x}}_{N}$ and $\bar{\bar{x}}$ are the column-, row- and overall means of ***X***, respectively, and $\bar{z}$ the row means of ***Z***. The sequential L-PLS algorithm as given in [15]:

Choose a value of *α*(0≤*α*≤1)For *a*=1,…,*A*components 

**Figure 1:**
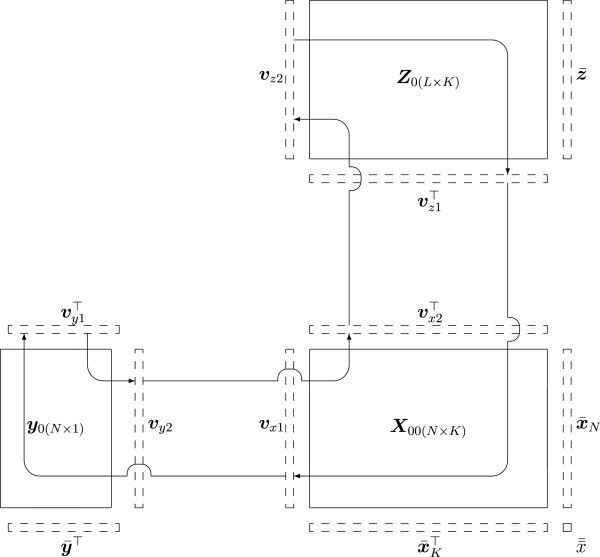
**L-PLS algorithm.** Steps for extracting the first set of v-vectors for the L-PLS algorithm are visualized by arrows. Deflated data matrices replace the initially centered matrices at subsequent steps of v-vector extraction. The mean-vectors used for data centering are also displayed.

Find latent v-vectors $v_{x2}^{a}$ (*K*×1) and $v_{z1}^{a}$ (*K*×1) by the NIPALS algorithm as shown in Figure 1, cycling through ***y***_*a*−1_, ***X***_*a*−1,*a*−1_and ***Z***_*a*−1_.

For chosen *α* set ***w***_*x*_ as a linear combination of $v_{z1}^{a}$ and $v_{x2}^{a}$ and normalize to length equal to 1.

(4) $$\begin{array}{lll} w_{x}^{a} & =\alpha v_{z1}^{a}+(1-\alpha )v_{x2}^{a}\; & \;\\ w_{x}^{a} & \leftarrow w_{x}^{a}/||w_{x}^{a}||\; & \; \end{array}$$

Construct score-vectors for ***X***and ***Z***, as:

(5) $$\begin{array}{lll} t_{x}^{a} & =X_{a-1,a-1}w_{x}^{a}\; & \;\\ t_{z}^{a} & =w_{x}^{a}\; & \; \end{array}$$

Let $T_{x}=(t_{x}^{1},\ldots ,t_{x}^{a})$ and $T_{z}=(t_{z}^{1},\ldots ,t_{z}^{a})$ (=***W***_*x*_). That is, the weights for ***X*** are used as scores for ***Z***.

Compute ***Y***–, ***X***– and ***Z***– loadings

(6) $$\begin{array}{lll} p_{y}^{a} & =y_{a-1}^{⊤}t_{x}^{a}{\left({t_{x}^{a}}^{⊤}t_{x}^{a}\right)}^{-1}\; & \;\\ p_{x}^{a} & =X_{a-1,a-1}^{⊤}t_{x}^{a}{\left({t_{x}^{a}}^{⊤}t_{x}^{a}\right)}^{-1}\; & \;\\ p_{z}^{a} & =Z_{a-1}t_{z}^{a}{\left({t_{z}^{a}}^{⊤}t_{z}^{a}\right)}^{-1}\; & \; \end{array}$$

Let $P_{y}=(p_{y}^{1},\ldots ,p_{y}^{a})$, $P_{x}=(p_{x}^{1},\ldots ,p_{x}^{a})$ and $P_{z}=(p_{z}^{1},\ldots ,p_{z}^{a})$.

Deflate the data matrices to form residual matrices

(7) $$\begin{array}{lll} y_{a} & =y_{a-1}-t_{x}^{a}{p_{y}^{a}}^{⊤}\; & \;\\ X_{a,a} & =X_{a-1,a-1}-t_{x}^{a}{p_{x}^{a}}^{⊤}\; & \;\\ Z_{a} & =Z_{a-1}-p_{z}^{a}{t_{z}^{a}}^{⊤}\; & \; \end{array}$$

end

In step 1) above *α*can be determined through cross-validation and the data driven choice of *α*indicates the relevancy of background information in modeling the genotype-phenotype relation. A large value of *α* indicates that background information, ***Z***, is highly relevant for explaining genotype-phenotype relations.

In essence, the L-PLS estimate of the regression coefficients for the above given model based on *A*components can be derived from the weights and loadings by:

(8) $$\hat{\beta }=W_{x}{\left(P_{x}^{⊤}W_{x}\right)}^{-1}P_{y}$$

#### Two stage variable elimination

Selection of variables based on Variables Importance on Projection (VIP) [24] is an accepted approach in PLS. We [17] recently suggested a stepwise estimation algorithm for parsimonious variable selection, where a consistency based variable selection procedure is adopted, and data has been split randomly in a predefined number of subsets (test and training). For each split, a stepwise procedure is adopted to select the variables. Stable variables that are being selected by stepwise elimination from all split of data are selected finally. This algorithm was also implemented here, but multivariate feature selection was performed in two distinct stages, first for selection of genotype variables from ***X***and then for the selection of background information variables from ***Z***. In both cases, ‘the worst’ variables were iteratively eliminated using a greedy algorithm. The algorithm required the ranking of column-variables of ***X*** and row-variables of ***Z***. For this $\mathrm{VI}P_{X_{k}}$ and $\mathrm{VI}P_{Z_{l}}$ are defined, measuring the importance of the column-variable *k*of ***X*** and the row-variable *l*of ***Z***respectively.

(9) $$\;\mathrm{VI}P_{X_{k}}=\sqrt{K\overset{A}{\underset{a=1}{\sum }}\left[\left({p_{y}^{a}}^{⊤}p_{y}^{a}\right){\left(t_{x_{k}}^{a}/∥t_{x}^{a}∥\right)}^{2}\right]/\overset{A}{\underset{a=1}{\sum }}\left({p_{y}^{a}}^{⊤}p_{y}^{a}\right)}$$

and

(10) $$\;\mathrm{VI}P_{Z_{l}}=\sqrt{L\overset{A}{\underset{a=1}{\sum }}\left[\left({p_{y}^{a}}^{⊤}p_{y}^{a}\right){\left(t_{z_{l}}^{a}/∥t_{z}^{a}∥\right)}^{2}\right]/\overset{A}{\underset{a=1}{\sum }}\left({p_{y}^{a}}^{⊤}p_{y}^{a}\right)}$$

The *VIP* weights the contribution of each variable according to the variance explained by each PLS component, and presents a combined effect of all components. Variable *k*can be eliminated if $\mathrm{VI}P_{X_{k}}<u$ and similarly variable *l* can be eliminated if $\mathrm{VI}P_{Z_{l}}<u$ for some user-defined threshold *u*∈[0,*∞*).

The stepwise elimination algorithm can be sketched as follows: Let ***U***_0_=***X***. 

For iteration *g*run ***y***and ***U***_*g*_through cross validated L-PLS. The matrix ***U***_*g*_has *K*_*g*_columns, and we get the same number of criterion values, sorted in ascending order as $\mathrm{VI}P_{X_{\left(1\right)}},\ldots ,\mathrm{VI}P_{X_{\left(K_{g}\right)}}$.

There are *M*criterion values below the cutoff *u*. If *M*=0, terminate the elimination here.

Else, let *S*=⌈*fM*⌉ for some fraction *f*∈〈0,1]. Eliminate the variables corresponding to the *S*most extreme criterion values.

If there are still more than one variable left, let ***U***_*g* + 1_contain these variables, and return to 1).

**Figure 2:**
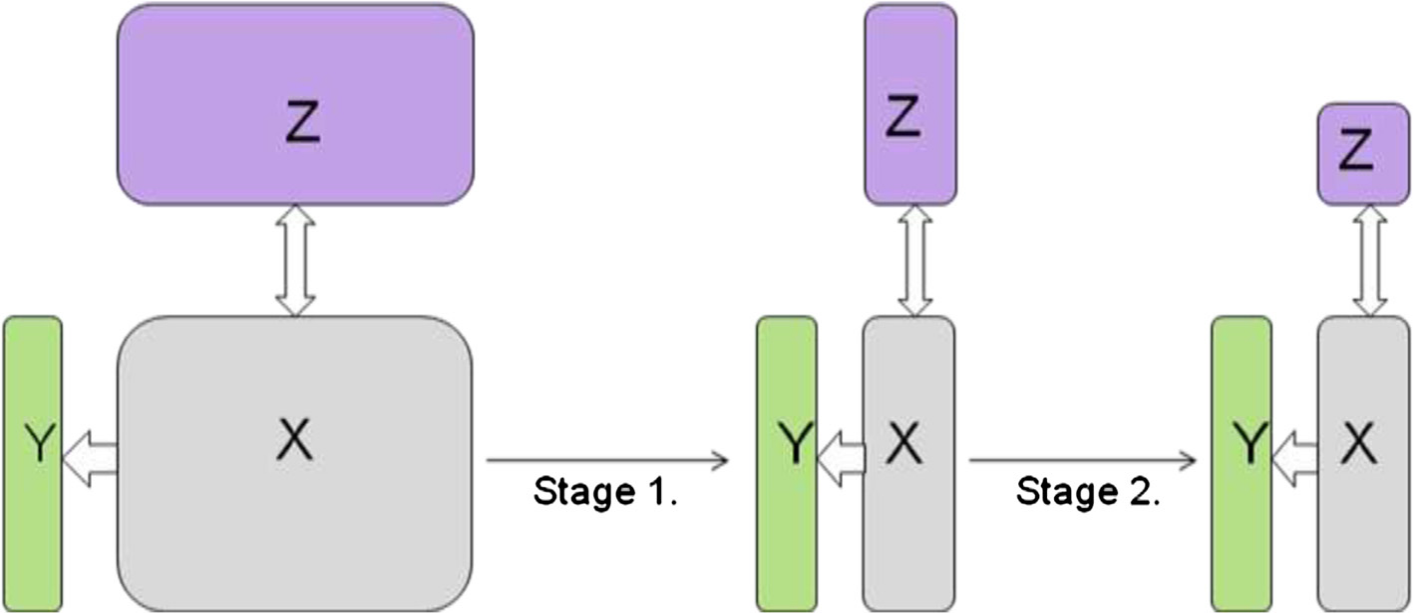
**An overview of variable elimination in two stages.** An overview of the variable elimination in two stages in L-PLS. Stage 1 eliminates variables in ***X***, using the *VIP*_*X*_criterion. After stage 1, we have reduced the number of columns (genes) in both ***X***and ***Z***. In stage 2, we eliminate rows of ***Z***, using the criterion *VIP*_*Z*_in a similar fashion.

The fraction *f*determines the ‘step length’ of the elimination algorithm, where an *f*close to 0 will only eliminate a few variables in every iteration. Elimination of variable in ***X***means an automatic elimination of the corresponding column variable in ***Z*** as well, because ***X*** and ***Z*** must have the same columns. Once the first stage elimination is completed, the above procedure can be repeated by considering ***U***_0_=***Z*** and $\mathrm{VI}P_{Z_{k}}$ for sorting row-variables of ***Z***for second stage elimination. An overview of variable elimination in both stages is given in Figure 2. The fraction *f* and threshold *u* can be obtained through cross validation. The fractions *u*can be obtained separately for each stage, but experiments revealed no big difference if we use the same value in both stages.

From each iteration *g*of the elimination, we get a cross validated root mean square error (RMSE) from training data, here denoted by *E*_*g*_. For both stages of the elimination, the number of influencing variables decreases at each iteration, and *E*_*g*_ will often decrease until some optimum is achieved, and then increase again as we keep on eliminating. A potentially much simpler model can be achieved by a relatively small increase in RMSE [17]. This means we need a rejection level *d*, where for each iteration beyond optimum root mean square error (RMSE) *E*^∗^ we can compute the t-test *p*-value between the optimum model response and the selected model response, to give a perspective on the trade-of between understandability of the model and the RMSE. Hence with a non-significant deviation from the optimum RMSE, a significant reduction in variables, and hence better understandability, can be achieved; for details, see [17].

### Choice of variable selection method for comparison

Two variable selection methods, where background information was not used in modeling, were compared with our suggested procedure, where background information was used in the modeling step. Hence,in total three models were considered, one was our suggested L-PLS with 2-stage stepwise elimination of genes and background information (M1), the second was ordinary PLS with stepwise elimination [17] of genes only (M2), and the third was a Soft-Thresholding PLS (ST-PLS) [3] (M3). We have recently used the latter approach for mapping of genotype to phenotype information [4].

All methods were implemented in the R computing environment http://www.r-project.org/.

#### The split of data into test and training and parameter tuning

**Figure 3:**
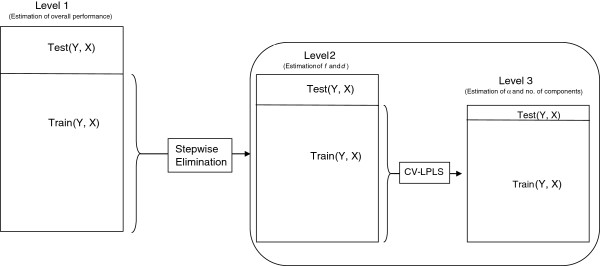
**An overview of the testing/training.** An overview of the testing/training procedure used in this study. The rectangles illustrate the predictor matrix. At level 1 we split the data into a test set and a training set (25/75). This was repeated 10 times. Inside our suggested method, the stepwise elimination, there are two levels of cross-validation. First a 10-fold cross-validation was used to optimize selection parameters *f*and *d*. Second, a leave-one-out cross-validation was used to optimize the L-PLS parameters, such as *α*and the number of components.

In the proposed algorithm it is possible to eliminate non-influential variables based on some threshold on *u* and also based on the statistical significance of *d*. Fixing the threshold *u*a priori could affect the performance of the algorithm. Thus, we performed variable elimination exclusively based on *d*, obtaining an optimum when using an upper limit of *u*=10. We also considered three step lengths (*f*=(0*.*1,0*.*5,1)). In the first regularization step, we tried different rejection levels (*d*=(0*.*80,0*.*90,0*.*95,0*.*99); hence, in each iteration, variable elimination was based on statistical significance. Model performance was computed in the L-PLS fitting and regularized elimination fitting stages. For accurate model estimation and to avoid over fitting, the data was split at three levels. Figure 3 gives a graphical overview of the procedure. At level 1, we split the data into a test set containing 25% of the genomes and a training set containing the remaining 75%. This split was repeated 10 times, each time sampling the test set at random, i.e. the 10 test and training sets were partially overlapping. These splits were used for model evaluation, where in each of the 10 instances, selected variables were used for classifying the level 1 test set, and RMSE was computed. In the regularized elimination fitting, there are two levels of cross-validation, as indicated by the right section of Figure 3. First, a 10-fold cross-validation was used to optimize the fraction *f* and the rejection level *d*in the elimination part of our algorithm. Second, at the final stage, leave-one-out cross-validation was used to estimate all parameters in the L-PLS method. These two procedures together corresponds to a comprehensive ‘cross-model validation’ [17,25]. Note that the above split of the data was done on ***y*** and ***X***, while the full ***Z***was used. Also note that this approach should not be used if dealing with very small sample sizes. In this situation, it is preferable to use predefined parameters.

## Results and discussion

### Simulated data

**Figure 4:**
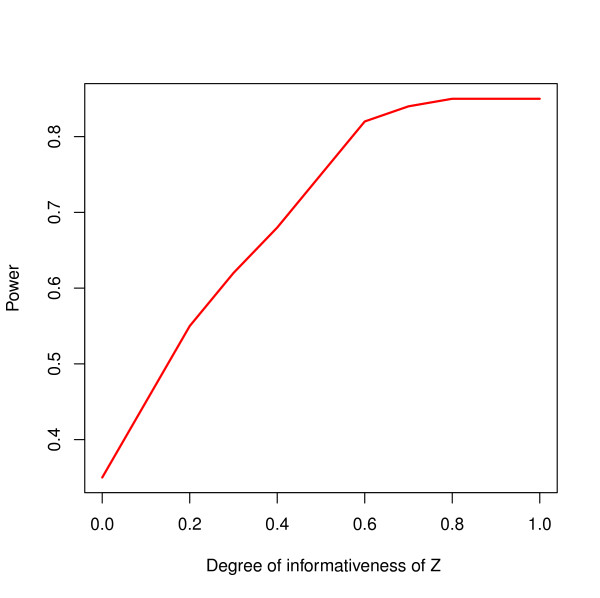
**Power analysis based on simulated data.** Power of selecting the correct variables as function distribution of degree of information content of Z in L-PLS *α*is presented. *α*=0 indicates the no background information is used in modeling, while higher value of *α*indicates the higher influence of background variables in modeling.

For the simulation data, a power analysis was conducted and results are presented in Figure 4. Here, the power of selecting the correct variables as function of the information content of ***Z*** in L-PLS *α*is presented. The power analysis shows that as the information content of ***Z*** in L-PLS i.e. *α*increases the ability to select the relevant variables also increases.

### Real data

**Figure 5:**
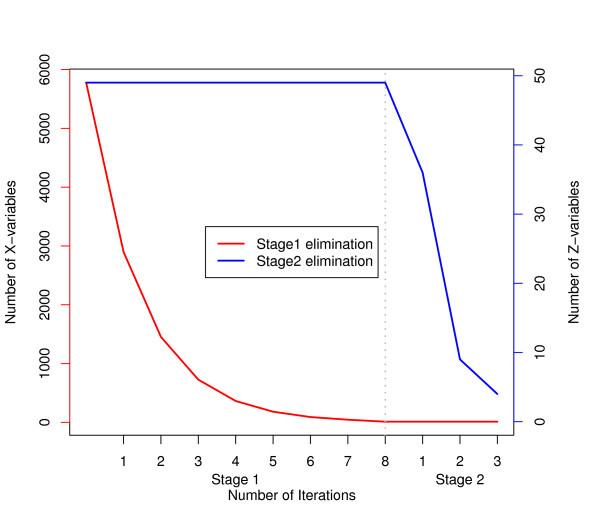
**An example of selection of variables in both stages.** Number of X- and Z- variables remaining in the model, after each iteration, for the response ‘Melibiose 2% Rate’. X-variables are displayed with red curve and are scaled on vertical left axis, while Z-variables are displayed with blue curve and are scaled on vertical right axis, both stages are shown on x-axis separately.

**Figure 6:**
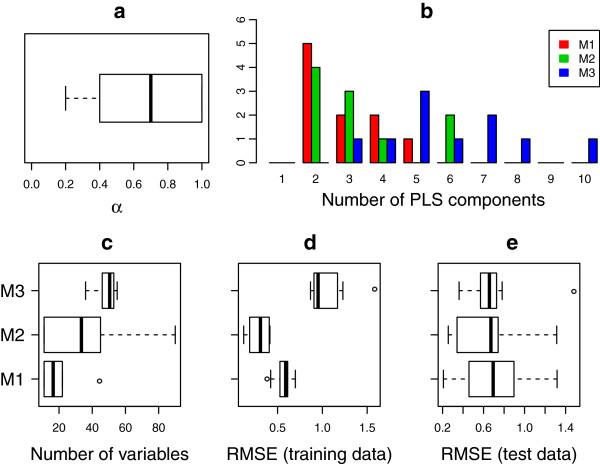
**The distribution of degree of informativeness of Z in L-PLS and comparison of complexity of the models, number of variables and RMSE.** Results for the phenotype ‘Melibiose 2% Rate’ are presented for three models, M1 (2-stage stepwise variable elimination in L-PLS), M2 (stepwise variable elimination in PLS) and M3 (St-PLS). In the upper left panel **(a)**, the information content of the background information (***Z***) in M1 is presented. Comparison of number of used PLS components in the upper right panel **(b)**, the number of selected variables in the lower left panel **(c)**, RMSE on training data in the lower center panel **(d)**, RMSE on test data in the lower right panel **(e)** for each model is presented.

To study the impact of background information on explanatory variables for genotype-phenotype relations in yeast, a 2-stage stepwise backward elimination procedure in L-PLS was used. We modelled each phenotype separately. The algorithm was illustrated using Melibiose Rate as an example. However, the performance was very similar also for other responses (phenotypes), as presented in Table 1. In total, we fitted 20 models, one for each phenotype. First, a genotype predictor matrix was derived by blasting the genes of each genome to a *S. cerevisiae* reference genome, and the best hit scores were used as numerical inputs to a genotype matrix [4]. Gene ontology terms, reflecting functional relatedness with regards to the gene product participation in similar molecular processes, together with data on gene dispensability (essential/not essential) and data on the number of gene paralogs present in founder genome, were used as background information. This data essentially reflects gene relationships in the S288C reference genome; relationships which may or may not be conserved in the species as a whole. We also included population genomic data reflecting the presence or absence, in each specific strain, of genetic variations with a potentially large impact on phenotypes. Gene copy number variations reflect potential gain-of-function mutations in particular lineages, whereas frameshift and premature stop codon mutations reflect potential loss-of-function mutations in respective lineages. The proposed model was fitted to each of the 20 phenotypes, and results are summarized in Table 1. Figure 5 exemplifies the progression of the 2-stage variable elimination for the phenotype Melibiose 2% Rate, representing the rate of growth of the set of yeast strains when supplied with carbon exclusively in the form of the melibiose. The number of genotype variables, ***X***, and background information, ***Z***, remaining after each iteration is given in the figure. In the first stage, variable reduction with respect to ***X***, was carried out in eight iterations. In the second stage, the remaining three iterations eliminates the variables in***Z***. We refer to this procedure, including both gene and background information, in an L-PLS approach, as Method 1 (M1). We compared M1 to a similar PLS approach, M2, which implements stepwise variable elimination on genes, with no background information included, and M3 *i.e.* ST-PLS, again on genes exclusively with no background information utilized. Hence, M1 utilizes background information in the modeling while M2 and M3 do not. In Figure 6, the distribution of the information content of ***Z***, indicating to what extent ***Z***is relevant for explaining genotype-phenotype relations, in M1 is presented, together with a comparison of the complexity of the models, the number of selected variables and the root mean square error on training and test data. For each split of the data, the information content of ***Z*** in M1, the number of used components and the number of X-variables were obtained. In the upper left panel, the degree of influence of ***Z***matrix in mapping genotype-phenotype relations is presented. The degree of influence, *α*, range from 0 to 1 where a higher value indicates a high influence of background information in genotype-phenotype mapping. With an average *α*=0*.*7, this indicates that the background information, in general, have a very considerable impact on genotype-phenotype mapping. In the top right panel, we see that the genotype-phenotype mapping, when applied using the stepwise elimination procedure adopted in M1 and M2, requires a lower number of PLS components than M3 to explain the phenotype pattern. Hence, M1 and M2 constitute less complex models than M3, because M3 ends with a higher number of components and a higher number of chosen variables. The lower left panel indicates that M1 selects a significantly lower number of genes for the genotype-phenotype mapping than M2 and M3. This means that noise, in terms of genes that do not actually contribute to explaining the phenotype, is substantially reduced when background information is included in the modeling step. The lower center panel shows that for the training data there was no significant difference in RMSE between M1 and M2, but both were lower than the RMSE for M3 (*p*<0*.*1). When applied on test data, all methods resulted in acceptable and similar RMSE, indicating that overall methods perform equally well (Figure 6e), lower right panel). However, M1 could achieve this performance using a much smaller number of variables. The number of variables required is a measure of the understandability of the model; hence, we conclude that M1, including background information in the PLS modeling, should allow for easier and more straight-forward interpretation of results. 

**Table 1:** An overview of model parameters and complexity

**Phenotype**	**f**	**d**	***α***	**No. of**	**RMSE**	**No. of selected**	**No. of selected**
				**components**		**genes**	**background variables**
Melibiose 2% Rate	0.1	0.99	0.73	5	0.61	30	15
Melibiose 2% Efficiency	0.1	0.99	0.65	4	0.60	30	14
Cupper chloride 0.375mM Rate	0.5	0.99	0.78	6	0.57	31	15
Cupper chloride 0.375mM Efficiency	0.1	0.90	0.51	9	0.52	30	14
NaCl 0.85M Rate	0.1	0.95	0.62	1	0.61	31	14
NaCl 1.25M Rate	0.5	0.95	0.85	2	0.80	30	15
NaCl 0.85M Efficiency	0.1	0.95	0.77	4	0.71	30	15
NaCl 1.25M Efficiency	0.1	0.99	0.67	5	0.60	30	15
Maltose 2% Rate	0.1	0.99	0.63	5	0.60	31	15
Maltose 2% Efficiency	0.1	0.90	0.54	7	0.50	30	15
Galactose 2% Rate	0.1	0.95	0.75	7	0.50	30	16
Galactose 2% Efficiency	0.1	0.95	0.56	7	0.61	30	15
Heat 37^*o*^*C* Rate	0.1	0.99	0.65	6	0.58	30	15
Heat 40^*o*^*C* Rate	0.1	0.99	0.78	6	0.82	30	15
Heat 37^*o*^*C* Efficiency	0.1	0.99	0.51	1	0.59	31	14
Heat 40^*o*^*C* Efficiency	0.5	0.90	0.62	8	0.67	30	15
Sodium arsenite oxide 3.5mM Rate	0.1	0.90	0.59	8	0.54	30	15
Sodium arsenite oxide 5mM Rate	0.1	0.99	0.66	5	0.62	30	15
Sodium arsenite oxide 3.5mM Efficiency	0.1	0.99	0.73	3	0.55	31	14
Sodium arsenite oxide 5mM Efficiency	0.1	0.90	0.51	2	0.63	31	14

The suggested approach select the model parameters at each level of 10-fold cross validation, hence the measure of central tendency is used to summaries the results. For each fitted model mode of selected step length (f), mode of rejection level (d), mode of model complexity (*α*and no. of components), mean of RMSE on test data, number of selected genes and number of selected background variables are listed.

**Figure 7:**
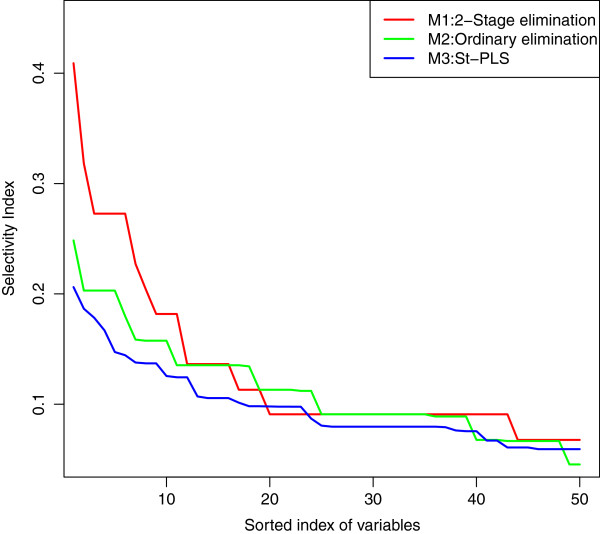
**Selectivity score.** The selectivity score is sorted in descending order and is presented here for X-variables (genes) for M1, M2 and M3. Only the first 50 values are shown from ***X***while all 51 values are shown from ***Z***.

A key requirement of any multivariate analysis is the stability and selectivity of the results. To evaluate model stability and selectivity, we [17] recently introduced a simple *selectivity score*: if a variable is selected as one out of *m* variables, it will get a score of 1/*m*. Repeating the selection for each split of the data, we simply add up the scores for each variable. Thus, a variable having a large selectivity score tends to be repeatedly selected as one among a few variables. In Figure 7, the selectivity score is sorted in descending order and is presented for X-variables (genes) in the upper left panel for M1, the upper right panel for M2 and the lower left panel for M3. The selectivity score indicating the stability of the selected Z-variables (GO- terms) obtained from M1 is presented in the lower left panel. M1 indeed selected many genes in a stable way, which is a fundamental requirement for any further analysis. A selectivity score above 0.2 for X-variables and above 0.06 for Z-variables is significantly larger than similar scores obtained by repeated fitting of models using random permutation on the phenotypes. Since traits are controlled by subsets of distinct genes [4], and some genes in the genome are of overall importance for handling variations in the external environment and affect a disproportionate number of phenotypes [26], we expect any method extracting relevant biological information to have a higher selectivity score than any random selection of genes. This was indeed the case for our proposed method M1. In fact, using the two-step L-PLS procedure, only 30 genes were selected from M1, corresponding to substantially higher selectivity than M2 and M3. Not surprisingly, these genes OLI1, YEH1, ATP8, PSY3, IFM1, SUV3, CAR1, ERG6, ILS1, YDR374C, SHO1, YDR476C, GLO3, APL5, RIX1, GPR1, VAR1, TTI2, YLR410WB, YDL211C, YDL218W, EHD3, MRPL28, RPT6, COX17, STE11, SUR4, YAP1, MRPL39, YNL320W, were involved in cellular functions directly relating to variations in the environment: transport, stress response, response to chemical stimulus and metabolism. They also tended to be affected by both strong loss-of-function (premature stop codons, frameshifts) and gain-of-function (copy number variation) mutations, as presented in Table 2. We found 72.% genes overlap between M1 and M2 and 67.9% genes overlap between M1 and M3 for Melibiose 2% Rate. The selection of background variables can be missed if only a few of the corresponding genes are significant [14], but the powerful structure of L-PLS, coupled with 2-stage stepwise elimination procedure, yields a stable list of genes and background information variables, which maps the genotype-phenotype relation. Finally, we have listed the mapped background information and genes for all 20 phenotypes in Table 1 and Additional file 1: Table S1 respectively.

**Table 2:** Selectivity score based selection of GO terms and gene variations

**Phenotypes**	**Influential gene variations and GO terms**
Melibiose 2% Rate	Paralog, frame shift variations, transport, stop codon variations, cellular protein catabolic process, transposition, copy number variations, response to stress, DNA metabolic process, mitochondrion organization, Essential.gene, RNA metabolic process, cellular amino acid and derivative metabolic process, response to chemical stimulus, response to chemical stimulus and metabolism
Melibiose 2% Efficiency	Copy number variations, transposition, Paralog, frame shift variations, stop codon variations, transport, cellular amino acid and derivative metabolic process, response to chemical stimulus, cell cycle, signal transduction, conjugation, RNA metabolic process, translation, mitochondrion organization, cellular carbohydrate metabolic process
Cupper chloride 0.375 mM Rate	Stop codon variations, Paralog, transposition, frame shift variations, copy number variations, RNA metabolic process, transport, protein modification process, response to stress, generation of precursor metabolites and energy, cellular respiration, DNA metabolic process, transcription, response to chemical stimulus, chromosome organization
Cupper chloride 0.375 mM Efficiency	Paralog, frame shift variations, transport, transposition, stop codon variations, Essential.gene, copy number variations, cellular amino acid and derivative metabolic process, RNA metabolic process, response to stress, protein modification process, chromosome organization, ribosome biogenesis, cell cycle, response to chemical stimulus
NaCl 0.85 M Rate	Generation of precursor metabolites and energy, cellular respiration, frame shift variations, stop codon variations, Paralog, copy number variations, transport, heterocycle metabolic process, sporulation resulting in formation of a cellular spore, transposition, transcription, cellular carbohydrate metabolic process, Essential.gene, RNA metabolic process, protein modification process
NaCl 1.25 M Rate	Cellular respiration, stop codon variations, frame shift variations, Paralog, generation of precursor metabolites and energy, Essential.gene, cellular lipid metabolic process, RNA metabolic process, transport, mitochondrion organization, cofactor metabolic process, transposition, response to chemical stimulus, transcription, DNA metabolic process
NaCl 0.85 M Efficiency	Paralog, transposition, transport, conjugation, frame shift variations, stop codon variations, signal transduction, RNA metabolic process, response to stress, chromosome organization, response to chemical stimulus, translation, ribosome biogenesis, mitochondrion organization, cellular amino acid and derivative metabolic process
NaCl 1.25 M Efficiency	Transposition, Paralog, copy number variations, frame shift variations, stop codon variations, response to stress, protein modification process, chromosome organization, transport, cellular amino acid and derivative metabolic process, translation, conjugation, RNA metabolic process, Essential.gene, mitochondrion organization
Maltose 2% Rate	Paralog, transposition, frame shift variations, stop codon variations, RNA metabolic process, response to chemical stimulus, transport, transcription, DNA metabolic process, copy number variations, response to stress, protein modification process, cellular amino acid and derivative metabolic process, heterocycle metabolic process, cellular aromatic compound metabolic process
Maltose 2% Efficiency	stop codon variations, generation of precursor metabolites and energy, Paralog, cellular amino acid and derivative metabolic process, transposition, cellular respiration, Essential.gene, protein modification process, heterocycle metabolic process, cellular aromatic compound metabolic process, transport, frame shift variations, RNA metabolic process, ribosome biogenesis, response to chemical stimulus
Galactose 2% Rate	DNA metabolic process, stop codon variations, translation, generation of precursor metabolites and energy, cellular respiration, Paralog, mitochondrion organization, copy number variations, cellular amino acid and derivative metabolic process, frame shift variations, transport, Essential.gene, response to stress, chromosome organization, meiosis
Galactose 2% Efficiency	Paralog, DNA metabolic process, frame shift variations, RNA metabolic process, stop codon variations, transport, generation of precursor metabolites and energy, cellular respiration, copy number variations, mitochondrion organization, cellular amino acid and derivative metabolic process, heterocycle metabolic process, transposition, protein folding, chromosome organization
Heat 37° Rate	Frame shift variations, transport, Paralog, generation of precursor metabolites and energy, heterocycle metabolic process, Essential.gene, cellular protein catabolic process, DNA metabolic process, mitochondrion organization, cellular respiration, transposition, stop codon variations, copy number variations, RNA metabolic process, transcription
Heat 40° Rate	Paralog, transport, frame shift variations, generation of precursor metabolites and energy, cellular protein catabolic process, heterocycle metabolic process, copy number variations, transposition, stop codon variations, DNA metabolic process, translation, response to stress, protein modification process, conjugation, Essential.gene
Heat 37° Efficiency	Generation of precursor metabolites and energy, DNA metabolic process, cellular amino acid and derivative metabolic process, cellular respiration, heterocycle metabolic process, stop codon variations, Essential.gene, RNA metabolic process, cofactor metabolic process, vitamin metabolic process, transposition, translation, Paralog, frame shift variations, transport
Heat 40° Efficiency	Paralog, frame shift variations, copy number variations, transport, transposition, protein modification process, cellular carbohydrate metabolic process, cellular amino acid and derivative metabolic process, heterocycle metabolic process, RNA metabolic process, response to stress, generation of precursor metabolites and energy, cell cycle, signal transduction, conjugation
Sodium arsenite oxide 3.5 mM Rate	Stop codon variations, Paralog, frame shift variations, copy number variations, cellular amino acid and derivative metabolic process, transposition, transport, response to stress, RNA metabolic process, conjugation, translation, transcription, protein modification process, Essential.gene, chromosome organization
Sodium arsenite oxide 5 mM Rate	Stop codon variations, copy number variations, transposition, frame shift variations, transport, generation of precursor metabolites and energy, cellular respiration, Paralog, protein modification process, Essential.gene, transcription, cell cycle, DNA metabolic process, response to chemical stimulus, ribosome biogenesis
Sodium arsenite oxide 3.5 mM Efficiency	Paralog, stop codon variations, frame shift variations, transposition, copy number variations, RNA metabolic process, cellular amino acid and derivative metabolic process, transport, DNA metabolic process, translation, cellular carbohydrate metabolic process, peroxisome organization, response to stress, protein modification process, transcription
Sodium arsenite oxide 5 mM Efficiency	Paralog, frame shift variations, stop codon variations, transposition, copy number variations, transport, protein modification process, cellular carbohydrate metabolic process, generation of precursor metabolites and energy, cellular respiration, Essential.gene, RNA metabolic process, response to stress, response to chemical stimulus, translation

Selected variables from the background information matrix ***Z***that have a selectivity score above 0.2 for each phenotype obtained through the proposed model. Variables correspond to the presence or absence of specific gene amplifications, and the presence or absence of premature stop codons and frameshifts.

Assuming that phenotypic variation within the species is controlled by either or both of lineage specific adaptive mutations, emerging as a consequence of lineage specific positive selection, or neutral variation, emerging as consequence of lineage specific relaxation of selective pressure that allow loss-of-function mutations to accumulate, we expected phenotype defining genes to show faster evolution than non-influential genes. This corresponds to a prediction of a higher ratio of nonsynonymous versus synonymous mutations since the split between *S. cerevisiae* and its closest relative *Saccharomyces paradoxus*[27]. Indeed, we found genes identified as influential through M1 to have been evolving 29% faster than non-influential genes (*p*<0*.*10). This indicates that these genes, as a group, have been subjected to either stronger positive selection or somewhat relaxed negative selection during the recent yeast history and supports that M1 extracts biologically relevant information.

## Conclusion

We have suggested the use of background information in the modeling step for genotype-phenotype mapping through L-PLS and a stepwise elimination procedure. We note that the derived results could give a slight decrease in RMSEP when background information is used, but more interestingly, this comes with more stability in the selection of variables (genes, GO terms and variations) used for genotype-phenotype mapping. We conclude that the approach is worth pursuing, and future investigations should be made to improve the computations of genotype signals, and variable selection procedure within the PLS framework.

## Competing interests

The authors declare that they have no competing interests.

## Authors’ contributions

TM initiated the project and the ideas. SS has been involved in the later development of the approach and the final algorithm. TM has done the programming and computations, with assistance from SS. TM has drafted the manuscript, with inputs SS, JW and LS. All authors have read and approved the final manuscript.

## Supplementary Material

Additional file 1 **Table S1.** Selectivity score based selected genes. Genes selected for each phenotype for genotype phenotype mapping by using 2-stage variable elimination and having selectivity score above 0.06. Click here for file

## References

[B1] Liti G Carter DM Moses AM Warringer J Parts L James SA Davey RP Roberts IN Burt A Koufopanou V Tsai IJ Bergman CM Bensasson D O’Kelly MJT van Oudenaarden A Barton DBH Bailes E Nguyen AN Jones M Quail MA Goodhead I Sims S Smith F Blomberg A Durbin R Louis EJ Population genomics of domestic and wild yeasts Nature 2009 458 337 341 10.1038/nature07743 19212322 PMC2659681

[B2] Warringer J Zörgö E Cubillos F Zia A Gjuvsland A Simpson J Forsmark A Durbin R Omholt S Louis E Trait variation in yeast is defined by population history PLoS Genet 2011 7 6 e1002111 10.1371/journal.pgen.1002111 21698134 PMC3116910

[B3] Sæbø S Almøy T Aarøe J Aastveit AH ST-PLS: a multi-dimensional nearest shrunken centroid type classifier via PLS J Chemometrics 2007 20 54 62

[B4] Mehmood T Martens H Saebo S Warringer J Snipen L Mining for genotype-phenotype relations in saccharomyces using partial least squares BMC Bioinformatics 2011 12 318 10.1186/1471-2105-12-318 21812956 PMC3175482

[B5] Badano A Modeling the bidirectional reflectance of emissive displays Appl Opt 2002 41 3847 3852 10.1364/AO.41.003847 12099591

[B6] Allison DB Thiel B Jean PS Elston RC Infante MC Schork NJ Multiple phenotype modeling in gene-mapping studies of quantitative traits: power advantages Am J Hum Genet 1998 63 1190 1201 10.1086/302038 9758596 PMC1377471

[B7] Kraft P de Andrade M Group 6: Pleiotropy and multivariate analysis Genet Epidemiol 2003 25 Suppl 1 S50—S56 14635169 10.1002/gepi.10284

[B8] Giaever G Flaherty P Kumm J Proctor M Nislow C Jaramillo DF Chu AM Jordan MI Arkin AP Davis RW Chemogenomic profiling: identifying the functional interactions of small molecules in yeast Nat Acad Sci 2004 101 793 798 10.1073/pnas.0307490100 PMC321760 14718668

[B9] Parsons AB Lopez A Givoni IE Williams DE Gray CA Porter J Chua G Sopko R Brost RL Ho CH Wang J Ketela T Brenner C Brill JA Fernandez GE Lorenz TC Payne GS Ishihara S Ohya Y Andrews B Hughes TR Frey BJ Graham TR Andersen RJ Boone C Exploring the mode-of-action of bioactive compounds by chemical-genetic profiling in yeast Cell 2006 126 611 625 10.1016/j.cell.2006.06.040 16901791

[B10] Chun H Keleş S Sparse partial least squares regression for simultaneous dimension reduction and variable selection J R Stat Soc: Ser B (Statistical Methodology) 2010 72 3 25 10.1111/j.1467-9868.2009.00723.x PMC2810828 20107611

[B11] Wende A Huss J Schaeffer P Giguere V Kelly D PGC-1 coactivates PDK4 gene expression via the orphan nuclear receptor ERR: a mechanism for transcriptional control of muscle glucose metabolism Mol Cell Biol 2005 25 10684 10694 10.1128/MCB.25.24.10684-10694.2005 16314495 PMC1316952

[B12] Jorgensen K Hjelle S Oye O Puntervoll P Reikvam H Skavland J Anderssen E Bruserud O Gjertsen B Untangling the intracellular signalling network in cancer-a strategy for data integration in acute myeloid leukaemia J Proteomics 2011 74 3 269 281 10.1016/j.jprot.2010.11.003 21075225

[B13] Mootha V Lindgren C Eriksson K Subramanian A Sihag S Lehar J Puigserver P Carlsson E Ridderstrale M Laurila E PGC-1alpha-responsive genes involved in oxidative phosphorylation are coordinately downregulated in human diabetes Nat Genet 2003 34 3 267 273 10.1038/ng1180 12808457

[B14] Tomfohr J Lu J Kepler T Pathway level analysis of gene expression using singular value decomposition BMC Bioinformatics 2005 6 225 10.1186/1471-2105-6-225 16156896 PMC1261155

[B15] Sæbø S Almøy T Flatberg A Aastveit A Martens H LPLS-regression: a method for prediction and classification under the influence of background information on predictor variables Chemometrics Intell Lab Syst 2008 91 2 121 132 10.1016/j.chemolab.2007.10.006

[B16] Vinzi V Chin W Henseler J Handbook of Partial Least Squares: Concepts, Methods and Applications 2010 Springer

[B17] Mehmood T Martens H Sæbø S Warringer J Snipen L A partial least squares based algorithm for parsimonious variable selection Algorithms Mol Biol 2011 6 27 38 10.1186/1748-7188-6-27 22142365 PMC3287970

[B18] Liti G Louis EJ Yeast evolution and comparative genomics Annu Rev Microbiol 2005 59 135 153 10.1146/annurev.micro.59.030804.121400 15877535

[B19] Warringer J Zorgo E Cubillos FA Gjuvsland A Louis EJ Omholt S Liti G Moses A Blomberg A Trait variation in yeast is defined by population history PLoS Genet 2011 7 1 15 10.1371/journal.pgen.1002111 PMC3116910 21698134

[B20] Warringer J Anevski D Liu B Blomberg A Chemogenetic fingerprinting by analysis of cellular growth dynamics BMC Chem Biol 2008 8 3 10.1186/1472-6769-8-3 18721464 PMC2532679

[B21] Dimmer EC Huntley RP Barrell DG Binns D Draghici S Camon EB Hubank M Talmud PJ Apweiler R Lovering RC The gene ontology - providing a functional role in proteomic studies Proteomics 2008 8

[B22] Martens H Anderssen E Flatberg A Gidskehaug L Høy M Westad F Thybo A Martens M Regression of a data matrix on descriptors of both its rows and of its columns via latent variables: L-PLSR Comput Stat Data Anal 2005 48 103 123 10.1016/j.csda.2003.10.004

[B23] Eriksson L Damborsky J Earll M Johansson E Trygg J Wold S Three-block bi-focal PLS (3BIF-PLS) and its application in QSAR SAR QSAR Environ Res 2004 15 5-6 481 499 10.1080/10629360412331297452 15669704

[B24] Nemeth M Multi-and megavariate data analysis Technometrics 2003 45 4 362 362

[B25] Anderssen E Dyrstad K Westad F Martens H Reducing over-optimism in variable selection by cross-model validation Chemometrics Intell Lab Syst 2006 84 1-2 69 74 10.1016/j.chemolab.2006.04.021

[B26] Hillenmeyer M Identifying relationships between genes and small molecules, from yeast to humans PhD thesis 2009 USA: Stanford University

[B27] Wall D Hirsh A Fraser H Kumm J Giaever G Eisen M Feldman M Functional genomic analysis of the rates of protein evolution Proc Nat Acad Sci USA 2005 102 15 5483 10.1073/pnas.0501761102 15800036 PMC555735

