# Supplementary material for: Improving stability and understandability of genotype-phenotype mapping in Saccharomyces using regularized variable selection in L-PLS regression
Source: BMC Bioinformatics. 2012 Dec 8;13:327. doi: 10.1186/1471-2105-13-327 (PMC3598729; doi:10.1186/1471-2105-13-327)
Supplement: Additional file 1 — Table S1. Selectivity score based selected genes. Genes selected for each phenotype for genotype phenotype mapping by using 2-stage variable elimination and having selectivity score above 0.06. [file 1471-2105-13-327-S1.pdf]

**Additional file 1, Table S1 - Selectivity score based selected genes**

Genes selected for each phenotype for genotype phenotype mapping by using 2-stage variable elimination and having selectivity score above 0.06

| Phenotypes | Influential genes |
|---|---|
| Melibiose 2% Rate | OLI1, YEH1, ATP8, PSY3, IFM1, SUV3, CAR1, ERG6, ILS1, YDR374C, SHO1, YDR476C, GLO3, APL5, RIX1, GPR1, VAR1, TTI2, YLR410WB, YDL211C, YDL218W, EHD3, MRPL28, RPT6, COX17, STE11, SUR4, YAP1, MRPL39, YNL320W |
| Melibiose 2% Efficiency | COS2, YFL041WA, YFL051C, BIO2, VNX1, RBD2, YRF1-4, YTH1, PAU17, ISR1, RFC5, SLI15, YOL159C, COB, COX3, YBL029W, YBR287W, YDL218W, ARO3, PDS1, YDR115W, HMO1, MHR1, SUR2, YER156C, COG3, VPS73, TDA3, ENT3, NKP2 |
| Cupper chloride 0.375mM Rate | ATP8, CST6, PMT2, YPT1, RMR1, DID2, FAL1, HDA2, IRE1, CDC45, LEU3, RSA1, RPC40, SGF29, NHP10, TFA2, YNL284CB, GAL4, YJL127CB, ECM27, AI5ALPHA, BI2, CCR4, SKT5, KTI11, CDC28, CTP1, YDR061W, RPL27B, TRS31 |
| Cupper chloride 0.375mM Efficiency | CTF19, DRS2, DTD1, MAL33, CST6, BI2, ILS1, NAS2, PSY3, MRPL39, SGF29, COS8, MET30, SGD1, GAL4, KNH1, SHO1, COS2, RSM24, YDR266C, RPT6, YHR131C, YJL043W, TTI2, YLL007C, UBR2, TRM12, PGA2, PEX17, RTC6 |
| NaCl 0.85M Rate | SUR2, ARH1, UBR2, YLR290C, WAR1, BTS1, YPL260W, OLI1, PET9, ATP16, YAP1, RIB2, CLB2, RTT107, TAF8, PMD1, SDS23, SNF6, SKN7, AI1, ATP6, SHP1, MEC1, YPC1, TRP1, ARO3, ASP1, TRS31, YCK3, RPS26B |
| NaCl 1.25M Rate | MRPL33, COB, OLI1, ATP16, RSM24, NUC1, CAP1, WAR1, BTS1, YPL260W, YJR012C, AI1, ETR1, ALG7, RMR1, TDA3, ECM25, ATP14, KAR3, SGF29, ATP8, CCR4, DRS2, CDC15, PRM9, COS2, VPS60, YAT2, RPL22B, ALG2 |

| | |
|---|---|
| NaCl 0.85M Efficiency | YAT2, PET123, TUF1, SUA7, SSA1, MRF1, FYV4, TPK1, TGL3, HRD1, YPL071C, RBD2, SAS3, SNF11, PRP42, SWI4, ZIP2, RTT107, BUD22, YTH1, OLI1, RPL19A, YDL211C, SEC20, BIM1, GLC7, CHD1, RIM15, INM1, RAD26 |
| NaCl 1.25M Efficiency | SPT4, YFL051C, SDS23, SUR4, LAP3, VNX1, TSR4, ERG10, YPL247C, IRE1, CST6, YIL082WA, PSF2, UTP13, RAD33, DAL82, NTG2, IBI1, SHC1, GDI1, YER190CB, YFL041WA, YFL042C, ATP8, OLI1, VAR1, SSA1, SSA3, ILS1, YDL218W |
| Maltose 2% Rate | AI3, YDR476C, NUC1, CAR1, YIL175W, ECM27, YEH1, PZF1, YIL174W, NIT2, ACF4, KTR4, DLD3, LAA1, CST6, CTF19, AOS1, SMX3, YBR255CA, YCR050C, TAT1, SHC1, GDI1, SCEI, ILS1, RCR2, GLO3, RNR2, TAD2, YJR056C |
| Maltose 2% Efficiency | SGF29, COB, KEX2, TRP1, NAS2, UFD4, LAP3, MAL33, MOH1, YFL051C, ATP8, OLI1, SLI15, PGA2, AI3, YDR476C, NUC1, LAC1, ATP14, TAT1, GDI1, AAC3, DAP2, SSA2, YET2, YNL024C, YOL036W, BI2, COX3, ADE1 |
| Galactose 2% Rate | YPT1, AI5ALPHA, PMD1, BI2, OLI1, PET9, AAC3, SDS23, TDA3, YLR132C, SUR4, UIP5, YFR012WA, AI1, AI2, ATP8, COB, BI3, VAR1, MCM2, ATP3, GRS1, ATP16, SIT4, YDL218W, ASP1, ECM10, GLO3, RPL22B, GTO1 |
| Galactose 2% Efficiency | PET9, AI5ALPHA, MSS1, AI1, TIF4631, YLR132C, RIA1, AOS1, YFR012WA, NIT2, YJR012C, SDS23, AIM45, RPO26, YJL127CB, AI5BETA, COB, BI4, VAR1, Q0255, MCM2, YBL029W, SSA3, BNA4, SCO1, AAC3, VAM6, THI3, RCR2, MAF1 |
| Heat $37^oC$ Rate | ATP8, PET9, ATP3, AIM45, OLI1, YAP1, CYT1, SEF1, ATP1, AAC3, DOS2, ASP1, ARH1, YPT1, RET2, SDS23, RPL15A, YLR126C, YLR290C, BTS1, YPL260W, AI5BETA, BI4, MCM2, YMC2, GRS1, SSE2, YBR242W, ILV6, THI3 |

| | |
|---|---|
| Heat 40$^oC$ Rate | ATP3, ATP8, OLI1, AIM45, PET9, ATP16, SGF29, TUB2, YLL032C, AHP1, YAP1, NBA1, YIL082WA, MOH1, VEL1, AI5ALPHA, GLO3, DOS2, BI4, MDH3, YDL218W, OCA6, OLE1, SDS23, CYR1, PET130, APS3, ALB1, ADE16, RPL15A |
| Heat 37$^oC$ Efficiency | AI1, OLI1, PET9, COX1, ATP8, PMD1, MRPL33, BI3, AAC3, PDC2, BUD20, SLK19, VPS20, PRC1, BUD17, HAP3, COB, RSM24, YET2, YCL019W, COX3, AI5BETA, ATP6, ADE16, SRN2, BIO4, SHG1, DAL80, Q0255, YBL095W |
| Heat 40$^oC$ Efficiency | CST6, ATP8, OLI1, SSA3, TTI2, YFL042C, YFL051C, ARO3, SUR4, TSR4, ERG10, RBD2, ZIP2, REC104, CKA1, YRF14, NOP12, CTF19, YTH1, SHC1, AI4, YDR476C, SDS23, YEH1, POP2, MRPS16, OXR1, YPL247C, SGF29, YCL019W |
| Sodium arsenite oxide 3.5mM Rate | YRF14, NOP12, YTH1, YPL247C, ATP8, MAL33, ZIP2, REC104, PSF2, RAD33, RPN7, MOH1, PHO5, SLI15, SHC1, SHO1, LAP3, SGF29, COB, BI2, BI3, SSA3, ILS1, RPL21A, COS2, YDL218W, GAL3, KRS1, TPI1, MRPL28 |
| Sodium arsenite oxide 5mM Rate | KTR4, YDR476C, YRF14, NOP12, YTH1, COB, COS2, DLD3, CKA1, CST6, SHO1, LIF1, RIF2, COX1, ATP8, ATP6, COX3, PET9, RSM24, ECM10, YCK3, GNA1, SDS23, YGL082W, YGL101W, YIP5, MLP1, ERG27, FMP27, ERG6 |
| Sodium arsenite oxide 3.5mM Efficiency | SGF29, CST6, DTD1, MET30, ULP1, SEF1, MAL33, CTF19, FIG1, DIA3, GPR1, SHC1, AI3, BI2, SCEI, SKT5, KTI11, RPS8A, PBY1, KTR4, VBA2, YDL218W, GAL3, YDR476C, ECM10, DLD3, GLC7, ERV14, YHR131C, NUC1 |
| Sodium arsenite oxide 5mM Efficiency | SGF29, KTR4, YDR476C, INN1, GAL3, CST6, CKA1, RIX7, ALK1, COB, TSC3, SKT5, COS2, DLD3, YHR131C, TRS31, RDS1, RSA3, PRM9, YBL059W, SSA3, NPL4, RTR2, RPL27B, SDS23, SER33, ADE16, SRN2, RIT1, PBI2 |
